# 'Peace of mind'? Demand for breast imaging investigation following normal clinical examination: establishing the patient benefits and service implications for a symptomatic service

**DOI:** 10.1186/bcr3804

**Published:** 2015-11-05

**Authors:** Miaad Al-Attar, Lubna Bashir, Lisa Grosvenor, Diane Lister, Elizabeth Denton, Moin Hoosein, Lakshmi Sundaram, Gayle McDonald, Niki Hartley

**Affiliations:** 1 Breast Care Centre, Glenfield Hospital, UHL, Leicester, UK

## Introduction

In the symptomatic service, we noted that requests for imaging after normal examination appeared to be significantly increasing but not improving cancer detection. We aimed to identify demand for imaging following normal clinical examination and their incidence of cancer.

## Methods

Our unit underwent clinic reorganisation, with the consultant surgeon as primary clinical assessor in February 2014. We carried out a retrospective audit, choosing a month prior and post service reorganisation. All patients referred to imaging with normal clinical examination (*P *= 1) were included. We recorded demographics, presenting complaint, requestor, findings and biopsies outcomes.

## Results

Pre consultant involvement, 576 patients were seen and 175 referred with *P *= 1 (30 %). A total of five biopsies (3 % of referred) were performed retrieving two malignancies (1 % of referred). Post reorganisation, 771 patients were seen and 308 referred to imaging with *P *= 1 (40 %). A total of 32 biopsies were performed (10 % of referred) with three malignancies (<1 % of referred). In this group only one patient was <40 years old. All cancers were invasive ductal (B5b). All malignancies were in areas of presenting concern. There was a significant increase in workload with decreasing sensitivity of radiology and clinical examination post reorganisation. The background incidence of malignancy was low and stable.

## Conclusion

There is increasing demand on imaging for patients with normal clinical examination unrelated to clinical seniority. Cancers are present even with normal clinical examination and patient's initial clinical concern proved to be an important predictor. Careful scrutiny of the patient's presenting symptom may allow detection of all cancers.

